# An examination and proposed theoretical model of risk and protective factors for bereavement outcomes for family members of individuals who engaged in medical aid in dying: A systematic review

**DOI:** 10.1177/02692163231172242

**Published:** 2023-05-02

**Authors:** Jonathan Singer, Courtney Daum, Amelia Evans, Sydnie Schneider, Margaret Vugrin, Elizabeth Loggers

**Affiliations:** 1 Department of Psychological Science, Texas Tech University, Lubbock, TX, USA; 2 Clinical Research Division, Fred Hutchinson Cancer Research Center, Seattle, WA, USA; 3 Libraries of the Health Sciences, Texas Tech University Health Science Center, Lubbock, TX, USA; 4 Division of Oncology, University of Washington, Seattle, WA, USA

**Keywords:** Medical Aid in Dying, bereavement, risk and protective factors, theoretical model, systematic review

## Abstract

**Background:**

Medical Aid in Dying is an end-of-life option that allows a physician to provide a patient with a prescription to end their life. Though Medical Aid in Dying intends to reduce suffering for a patient, opponents argue Medical Aid in Dying may increase suffering for the family members during bereavement. To better understand the bereavement outcomes for family members/friends following Medical Aid in Dying, an exhaustive review of the risk and protective factors for bereavement outcomes is warranted.

**Aim:**

This systemic review aimed to identify studies that examined bereavement outcomes of family members of individuals who engage in Medial Aid in Dying, identify risk and protective factors for bereavement outcomes, and propose a theoretical model to enhance conceptual clarity.

**Design:**

A mixed-method systematic review.

**Data sources:**

Ten databases were searched on June 16, 2021 and later conducted two updates (latest April 25, 2022).

**Results:**

Thirteen articles met inclusion criteria. Risk and protective factors were identified pre-Medical Aid in Dying and risk factors post-Medical Aid in Dying. Few studies compared bereavement outcomes for family members of individuals utilizing Medical Aid in Dying to family members who lost someone to natural loss.

**Conclusions:**

This study provides equivocal results about the effects of Medical Aid in Dying on family members following the loss. The theoretical model outlines potential risk and protective factors. This model provides a greater understanding of possible universal risk and protective factors for family members of individuals who engaged in Medical Aid in Dying.

**What is already known about the topic?**

- Current evidence suggests that Medical Aid in Dying intends to reduce suffering for a patient with a terminal illness.
- Opponents of Medical Aid in Dying argue it can increase suffering for the family members during bereavement.
- Differences exist in the literature regarding risk and protective factors for bereavement outcomes.

**What this paper adds**

- This systematic review of the literature identified equivocal results about the effects of Medical Aid in Dying on family members following the loss.
- Risk and protective factors were identified that occur pre-Medical Aid in Dying and risk factors were identified that occur post-Medical Aid in Dying.

**Implications for practice, theory or policy**

- This study provides practitioners with possible risk factors for poor bereavement outcomes for family members of persons who utilize Medical Aid in Dying.
- This study provides a theory driven model that will help guide future studies.

## Introduction

Medical Aid in Dying in the United States is an end-of-life option that allows a physician to provide an eligible patient with a prescription for a lethal dose of medication to end a person’s life when they have less than 6 months to live. Over the last decade, there has been an increase in the legalization of Medical Aid in Dying around the world, with more than 12 countries legalizing Medical Aid in Dying in at least one state or province as of June 2022.^1^ In the United States, 11 states have legalized Medical Aid in Dying. Though Medical Aid in Dying is intended to reduce suffering for a patient, opponents of Medical Aid in Dying legalization often argue that Medical Aid in Dying may increase suffering for family/friend of patients during bereavement, putting them at increased risk of prolonged grief disorder, post-traumatic stress disorder, and anxiety disorders.^2,3^ However, supporters of Medical Aid in Dying argue that having a family member/friend utilize Medical Aid in Dying can be beneficial for long term outcomes for bereaved family members.^2,3^

Limited research exists regarding the bereavement outcomes in family members of individuals who engage in Medical Aid in Dying. This is despite recent calls^1,4^ to examine bereavement outcomes, such as prolonged grief disorder, as well as risk and protective factors where Medical Aid in Dying is legal. Grief, and individuals own grief process, following the death of a family member is normal, but when grief reactions occur that result in loss of functioning over an extensive amount of time (i.e. a year or more) it can result in prolonged grief disorder, which has been recently added to the DSM-5-TR and the ICD-11.^5^ Current findings suggest bereavement risk and protective factors among family members of individuals who died due to natural causes or violent events include social support, mental health, and “preparedness for death.”^6^ Further, there is an abundance of research that has investigated the important role of attachment and pre-existing complex relationships on bereavement outcomes no matter the type of loss.^7,8^ Therefore, we expect many of these factors (i.e. attachment; pre-existing complex relationships; pre-death depression; ethnicity; burden) will impact bereavement for individuals whose family member engaged in Medical Aid in Dying.

In a systematic review of bereavement outcomes for individuals who lost a family/friend due to a violent event (e.g. homicide; suicide; accident), greater pre-death depression and worse emotional health prior to the death were related to worse bereavement outcomes (major depressive disorder; prolonged grief disorder).^5^ Similar findings exist for losses due to dementia. Furthermore, it is apparent from these systematic reviews that there are also risk and protective factors that are unique to the cause of death. For example, ethnicity (i.e. being Hispanic) and higher caregiver burden were significant predictors of poor bereavement outcomes for individuals who lost someone to dementia,^6^ but not for bereaved individuals whose family/friend died from a violent loss.^6^ These findings provide a foundation for the investigation of transdiagnostic and additional, unique risk and protective factors of bereavement outcomes in individuals whose family/friend has engaged in Medical Aid in Dying.

Medical Aid in Dying is typically requested after a long course of managing a terminal illness and has been characterized as a complex process that can include moral and ethical dilemmas regarding the patient and family’s decision to engage in Medical Aid in Dying. Researchers^2,3,9^ have stated the decision is not solely related to the amount of time following the diagnosis of the terminal illness but could be related to factors that emerged decades *before* the terminal illness (e.g. relationship satisfaction before the terminal diagnosis). In contrast, studies have also suggested there are specific risk factors experienced by the family member (e.g. pre-death grief) that occur *after* the patient is diagnosed with a terminal illness.^10–12^ Moreover, Medical Aid in Dying is often associated with increased stigma related to a patient’s or family member’s perceived morality of Medical Aid in Dying,^13^ which could both happen before (i.e. decision-making surrounding Medical Aid in Dying) and after the death (i.e. toward the family member during bereavement). Accordingly, when investigating factors related to bereavement outcomes of family members of individuals who have engaged in Medical Aid in Dying, it is imperative to delineate those risk and protective factors that may occur at various distinct stages of the illness process (i.e. following terminal diagnosis; *post-Medical Aid in Dying* periods).

## Methods

This systemic review aimed to (1) examine bereavement outcomes of family members of individuals who engaged in Medical Aid in Dying; (2) identify the existing literature on specific risk and protective factors for bereavement outcomes of aforementioned family members; (3) through data synthesis, propose a novel theoretical model, one which highlights risk and protective factors at each stage of the Medical Aid in Dying process (i.e. following terminal diagnosis; bereavement). See Appendix A for more details about the information sources used, search strategy, study records, and selection process.

### Systematic review design

This mixed-method systematic review and protocol were prospectively registered on Open Science Framework (created 5-18-2022). This study used a phenomenological approach in which we integrated findings of primary quantitative and qualitative studies to build a network of related concepts that together provide a comprehensive understanding of the effects of Medical Aid in Dying on bereavement outcomes in family members, including the risk and protective factors for these bereavement outcomes based on extant literature. See Appendix A for more details about the information sources used, search strategy, study records, and selection process.

### Search strategy and data sources

The search strategy was developed in consultation with a specialist librarian at Texas Tech University Health Science Center (MV), which has been implemented in past studies.^14,15^ We also utilized Preferred Reporting Items for Systematic Reviews and Meta-Analyses (PRISMA) guidelines.^16^ Ten databases were selected: PubMed (via National Library of Medicine’s PubMed.gov), Embase (via Elsevier’s Embase.com), Cochrane Central Register of Controlled Trials/Cochrane CENTRAL (via Wiley’s Cochrane Library), PsycINFO (via Ovid), Web of Science Core Collection (via Clarivate Analytics), Cumulative Index to Nursing and Allied Health Literature (CINAHL) (via EBSCO), PsycArticles (via EBSCO), Sociological Abstracts (via Proquest), PsychiatryOnline, and SCOPUS (via Elsevier’s Scopus.com). The terms that were used as part of the search strategy were “medical aid in dying,” or “MAID,” or “death with dignity,” or “assisted suicide” or “assisted death” or “medically assisted suicide” or “physician-assisted suicide” or “DWD” or “voluntary assisted dying” or “VAD” or “aid in dying” AND “end of life,” or “depression,” or “prolonged grief,” or “prolonged grief disorder,” or “complicated grief,” or “grief,” or “traumatic grief,” or “bereavement,” or “major depressive disorder,” or “mental health,” or “physician assisted suicide,” or “terminal illness” or “post-traumatic stress disorder.” Concepts were combined with the Boolean AND operator, and the Cochrane Handbook filter was used to exclude animal-only studies.^17^ The 10 databases were comprehensively searched on June 16, 2021. The first author (JS) later conducted two updates (the latest on April 25, 2022) and searched the 10 databases to identify if any new articles should be added to the review that were published after April 25, 2021. Results were entered as Research Information System files (i.e. standardized tag format) in Covidence, a web-based software platform for systematic review development.^18^

### Selection strategy

Articles were deemed eligible for inclusion if they evaluated, through qualitative or quantitative analysis, family member/friend’s bereavement outcomes (e.g. depression; grief; post-traumatic stress disorder) associated with individuals who had engaged in Medical Aid in Dying. Articles were also eligible if they examined risk and protective factors for bereavement outcomes for family members or friends of individuals who engaged in Medical Aid in Dying.

### Screening process

After duplicates were removed, abstracts were reviewed by two independent reviewers for initial eligibility. Articles were considered for full-text review if both reviewers (JS; CD) agreed they met inclusion criteria. When there was disagreement between two reviewers, discrepancies were decided by the senior author (ETL). All studies that met criteria for full-text evaluation were then independently reviewed by two reviewers and disagreements between reviewers were discussed with the first and second author. A standardized template was developed to extract pre-specified information from the final set of included articles. For each article, a reviewer (AE) completed the coding template to extract the pre-determined information from each article.

### Data synthesis and analysis

Studies that were selected were individually entered by the authors into an Excel table for data extraction. Authors (JS; AE) were instructed to include title, authors, whether the study was qualitative, quantitative, or mixed methods, design of the study, whether the study was prospective or retrospective, the purpose of the study, sample details (mean age, gender, race/ethnicity, relationship to patient/deceased, disease status), and key qualitative and quantitative findings. The first author (JS) organized the studies by qualitative, quantitative, or mixed design. Following organization, a thematic synthesis was conducted in order to evaluate risk and protective factors that were analyzed in each study. The synthesis began with “line-by-line” coding of the included articles (*n* = 13). These were then aggregated by both risk and protective factor and then by *pre-Medical Aid in Dying* and *post-Medical Aid in Dying.*

### Quality assessment

Two investigators (JS; AE) independently assessed the quality of the studies; there were no discrepancies in these assessments. The National Institute of Health Quality Assessment Tool for Case Control^19^ as used for all studies. Table 1 highlights the total score for quantitative studies with scores ranging from 0 to 12 with higher scores representing higher quality. The studies are then classified as “good,” “fair,” or “poor” by the reviewers. For qualitative studies, nine criterion variables were given a score between 1 (poor) and 4 (good)^20^ with overall scores ranging from 9 to 36. High quality was indicated by a score of 30–36, medium quality 24–29, and low quality 9–24.

**Table 1.** Articles examining risk and protective factors of bereavement outcomes in family members of individuals who engage in MAID.

Author et al.	Methods	Design	Purpose of the study	Illness context for family member	Sample characteristics	Relationship to patient/deceased	Outcome measures	Country study conducted	Study quality
Beuthin et al., 2021^b^	Qualitative	Cross Sectional	To understand the bereavement experience of family and friends of individuals who engaged in MAID	Terminal Illness (*n* = 9) Total: (*n* = 9)	Mean age: Not provided Gender: Not provided Race/Ethnicity: Not provided	Spouse (*n* = 4) Friend (*n* = 2) Daughter (*n* = 2) Sister (*n* = 1)	N/A	Canada	R1: Score: 28/36 Medium R2: Score: 28/36 Medium
Buchbinder et al., 2018^b^	Qualitative	Cross Sectional	To explore the experiences of lay caregivers involved with MAID in the U.S., focusing on the day of death.	Cancer (*n* = 10), ALS (*n* = 1)*** Total: (*n* = 11)	Mean age: 60.3 Gender: 17 female, 2 male Race/Ethnicity: Not provided	Friend (*n* = 11) Spouse/partner (*n* = 4) Adult Child (*n* = 3) Sibling (*n* = 1)	N/A	U.S.	R1: Score: 27/36 Medium R2: Score: 29/36 Medium
Frolic et al., 2020^b^	Qualitative	Cross Sectional	To explore the legacy of a MAID death for individuals who accompanied a loved one through the process	Terminal Illness (*n* = 14)*** Total: (*n* = 14)	Mean age: Not provided Gender: Not provided Race/Ethnicity: Not provided	Adult Child (*n* = 9) Spouse (*n* = 4) Friend (*n* = 2) Sibling (*n* = 1)	N/A	Canada	R1: Score: 26/36 Medium R2: Score: 27/36 Medium
Gamondi et al., 2020^b^	Qualitative	Cross Sectional	To explore how Swiss families interact with health care professionals and right-to-die associations regarding assisted suicide and their choices around disclosure	Cancer (*n* = 12) Other (*n* = 6)*** Total: (*n* = 18)	Mean age: 60 Gender: 11 men and 17 women Race/Ethnicity: Not provided	Spouse/partner (*n* = 8) Adult child (*n* = 7) Friend (*n* = 5) Other (*n* = 8)	N/A	Switzerland	R1: Score: 34/36 High R2: Score: 32/36 High
Ganzini et al., 2009^a^	Mixed	Cross Sectional	To describe how patient’s end-of-life choices affected family caregivers of Oregonians who requested aid in dying. Also, to measure severity of grief symptoms, use of mental health services, and depression in the family caregivers. The second goal was to compare these outcomes to those of family caregivers of decedent Oregonians who had not pursued hastened death.	Cancer (*n* = 68) ALS (*n* = 4) Other (*n* = 12)*** Comparison group Cancer (*n* = 50) ALS (*n* = 6) Other (*n* = 7)*** Total: (*n* = 118)	Mean age: 60.9 Gender: 29 Male, 66 Female Race/Ethnicity: 95 White/Caucasian Comparison Group Mean age: 60.1 Gender: 13 Male, 50 Female Race/Ethnicity: 61 White/Caucasian 2 Other	Spouse/partner (*n* = 52) Adult Child (*n* = 28) Other (*n* = 15) Comparison group Spouse/partner (*n* = 37) Adult Child (n=17) Other (*n* = 9)	Social Support: 16-item Interpersonal Support Evaluation List Prolonged grief: Complicated Grief-Revised (ICG-R) short form Depression: Beck Depression Inventory (BDI)	U.S.	R1: Score: 9/10 Good R2: Score: 9/10 Good
Hashemi et al., 2021^b^	Qualitative	Cross Sectional	To explore caregivers’ experience with MAID in the home-setting and their bereavement process	Cancer (*n* = 7) Neurodegenerative (*n* = 4) Parkinson’s (*n* = 1) Other (*n* = 1) Total: (*n* = 13)	Mean age: 74.4 Gender: 8 Male; 7 Females Race/Ethnicity: 13 White/Caucasian	Spouse/partner (*n* = 7) Child (*n* = 5) Other (*n* = 1)	IES-R Brief Grief	Canada	R1: Score: 29/36 Medium R2: Score: 29/36 Medium
Holmes et al., 2018^b^	Qualitative	Cross Sectional	To explore the experience of family and close friends of patients seeking MAID in Canada	Organ failure (*n* = 8) Neurologic (*n* = 5) Cancer (*n* = 4) Dementia (*n* = 1) Total: (*n* = 18)	Mean age: Not provided Gender: Not provided Race/Ethnicity: Not provided	Adult Child (*n* = 9) Spouse (*n* = 5) Friend (*n* = 3) Sibling (*n* = 1)	N/A	Canada	R1: Score: 32/36 High R2: Score: 32/36 High
Snijdewind et al., 2013^b^	Qualitative	Cross Sectional	To identify and categorize the characteristics of euthanasia and MAID requests	Cancer (*n* = 14) Dementia (*n* = 4) Other (*n* = 4) Total: (*n* = 22)	Mean age: Not provided Gender: 22 Females; 4 Males Race/Ethnicity: Not provided	Spouse (*n* = 10) Adult Child (*n* = 7) Other (*n* = 5)	N/A	Netherlands	R1: Score: 32/36 High R2: Score: 30/36 High
Srinivasan, 2019^b^	Qualitative	Cross Sectional	To explore bereavement experiences with an assisted death	Terminal Illness (*n* = 22) Total: (*n* = 22)	Mean age: Not provided Gender: 15 Women, 7 Men Race/Ethnicity: White/Caucasian = 100%	Spouses (*n* = 16) Other (*n* = 6)	N/A	U.S.	R1: Score: 24/36 Low R2: Score: 22/36 Low
Swarte et al., 2003^a^	Quantitative	Cross Sectional	To assess how MAID in terminally ill cancer patients affects the grief response of bereaved family and friends	Cancer (*n* = 58)*** Comparison group Natural Death (*n* = 114)*** Total: (*n* = 172)	Mean age: 48 Gender: 102 Women, 87 Men Race/Ethnicity: Not provided Comparison group Mean age: 49 Gender: 184 Women, 132 Men Race/Ethnicity: Not provided	Spouses (*n* = 33) Parent (*n* = 2) Adult Child (*n* = 30) Sibling (*n* = 14) Other (*n* = 37) Comparison group Spouses (*n* = 61) Parent (*n* = 4) Adult Child (*n* = 117) Sibling (*n* = 66) Other (*n* = 22)	Inventory of Traumatic Grief Texas revised inventory of grief Impact of Event Scale Symptom Checklist Depressive Adjective checklist	Netherlands	R1: Score: 9/10 Good R2: Score: 8/10 Good
Wagner et al., 2011a^a^	Quantitative	Cross Sectional	Evaluate the relationship between forensic investigations and mental health, specifically PTSD. Further, descriptively analyzed the behavior of the participating officials as perceived by a relative	Cancer (*n* = 44) Non-fatal age-related diseases (*n* = 31) Cardiac disease (*n* = 12) Alzheimer/dementia (*n* = 5) Mental Disorder (*n* = 3) Total: (*n* = 95)	Mean age: 60.15 Gender: 48 Women, 41 Men Race/Ethnicity: Not reported	Adult child (*n* = 40) Partner (*n* = 28) Parent (*n* = 2) Sibling/friend (*n* = 15)	Impact of Event Scale-Revised Inventory of Complicated Grief-SF, SCL-90; Forensic investigation experience scale	Switzerland	R1: Score: 9/10 Good R2: Score: 9/10 Good
Wagner et al., 2011b^a^	Quantitative	Cross Sectional	To examine the effects of perceived social acknowledgement on symptoms of post-traumatic stress and CG	Cancer (*n* = 44) Non-fatal age-related diseases (*n* = 31) Cardiac disease (*n* = 12) Alzheimer/dementia (*n* = 5) Mental Disorder (*n* = 3) Total: (*n* = 95)	Mean age: 60.15 Gender: 48 Women, 37 Men Race/Ethnicity: Not reported	Adult child (*n* = 40) Partner (*n* = 28) Parent (*n* = 2) Sibling/friend (*n* = 15)	Impact of Event Scale-Revised Inventory of Complicated Grief-SF, SCL-90, Social Acknowledgement as a Victim or Survivor Questionnaire	Switzerland	R1: Score: 9/10 Good R2: Score: 8/10 Good
Wagner et al., 2012a^a^	Quantitative	Cross Sectional	To examine the impact that witnessing assisted suicide has on the mental health of family members or close friends.	Cancer (*n* = 44) Non-fatal age-related diseases (*n* = 31) Cardiac disease (*n* = 12) Alzheimer/dementia (*n* = 5) Mental Disorder (*n* = 3) Total: (*n* = 95)	Mean age: 60.15 Gender: 48 Women, 37 Men Race/Ethnicity: Not reported	Adult child (*n* = 40) Partner (*n* = 28) Parent (*n* = 2) Sibling/friend (*n* = 15)	Impact of Event Scale-Revised Inventory of Complicated Grief-SF, SCL-90	Switzerland	R1: Score: 9/10 Good R2: Score: 8/10 Good

R1: Reviewer 1; R2: Reviewer 2.

a The National Institute of Health Quality Assessment Tool for Case Control (2017). Total score (0–12 depending on not applicable) and study is classified as “good,” “fair,” or “poor.”

b Hawker et al.^20^ quality assessment tool for qualitative studies. Nine questions from a range of 1 point (very poor) to 4 points (good). High quality, 30–36 points; medium quality, 24–29 points; low quality, 9–24 points.

*** There were multiple family members interviewed for a single patient.

## Results

**Figure 1.**
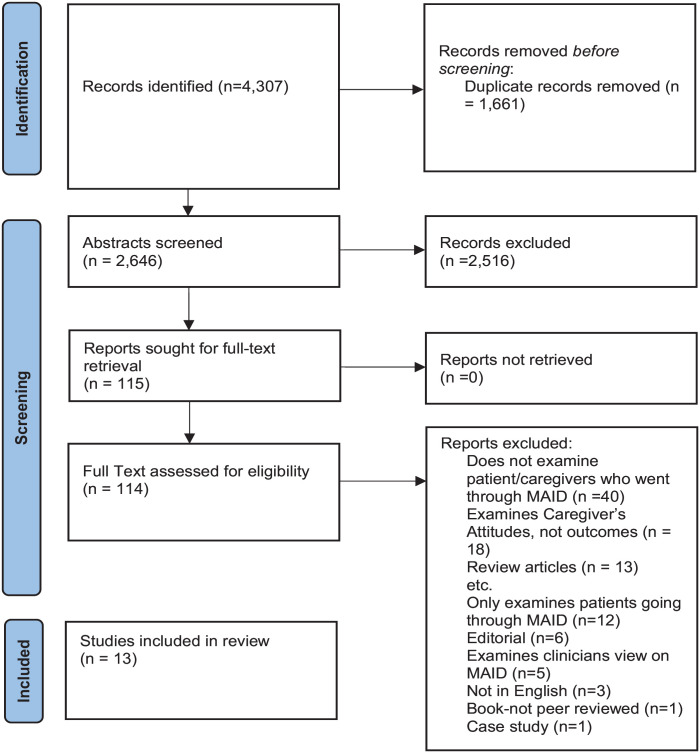
PRISMA flow diagram. Source: Page et al.^21^

A total of 4307 articles were reviewed. A final set of 13 full-text articles underwent qualitative synthesis (see Figure 1 for PRISMA). The studies were predominantly qualitative (*n* = 9). Three were quantitative and one was mixed methods. All of the studies were cross-sectional (*n* = 13). Most of these studies (*n* = 12) had mixed samples (i.e. eligibility criteria included any terminal diagnosis) for the person who engaged in Medical Aid in Dying. However, most patients had some form of cancer, as more than 10 of the 13 studies had at least a subset of participants endorsing cancer as the individual’s underlying diagnosis. Three studies stated that the participants had a terminal illness and did not specify the type of illness (see Table 1).

### Bereavement outcomes following Medical Aid in Dying

Four studies were either quantitative or mixed methods. Three of the studies^22–24^ used the same dataset of participants to investigate outcomes for bereaved individuals whose family member or friend engaged in Medical Aid in Dying. These three studies concluded bereaved family members or friends of individuals who engaged in Medical Aid in Dying experience poor outcomes during the bereavement process. For example, one study^24^ found that, in a sample of 85 bereaved family members and friends, 13% met criteria for post-traumatic stress disorder (PTSD), 6.5% met criteria for subthreshold PTSD, 4.9% met criteria for complicated grief, 16% met criteria for depression, and 6% met criteria for an anxiety disorder. However, this study did not have a comparison group to further contextualize study results. The fourth quantitative study^2^ included a comparison group (loss due to natural causes) and found there were no significant differences in negative bereavement outcomes (i.e. major depressive disorder, prolonged grief disorder, social support, mental health utilization, and utilization of hospice) following the loss, based on type of loss (i.e. Medical Aid in Dying vs natural causes).

### Protective factors

Across the 13 articles there were five primary themes identified as protective factors for negative bereavement outcomes. The five factors included preparedness for death, place of death, sense of control and autonomy, reduction in suffering/burden, and being able to support the patient’s end-of-life wishes. All themes identified were in at least two of the qualitative articles in this systematic review.

The first theme identified was preparedness for the death, which was found in eight of the 10 qualitative studies. Three subthemes were identified under preparedness: “saying goodbye,” preparedness, and feeling the loss was less of a shock. In regard to “saying goodbye,” two studies^25,26^ found that participants who reported being prepared for the death perceived that they were able to have certainty of the date and time of the death, which afforded them a “countdown” to say goodbye. Further, one study^27^ stated that participants reported feeling relief when they got to say goodbye on their terms. The second subtheme identified was preparedness, which allowed the bereaved individual time to grieve before the actual death as this is a final act at the end of a prolonged illness in which death is expected. Therefore, Medical Aid in Dying and the preparedness that comes with it, has been found to effect bereavement outcomes, which was shown in two studies.^25,27^ These studies found that engaging in Medical Aid in Dying allowed the family member time to “digest” the loss; due to the planned nature of Medical Aid in Dying, family members and friends could start their bereavement process earlier. The last subtheme was the Medical Aid in Dying process allowed the death to be less of a shock to participants (i.e. family; friends).^28^ Similar to the subtheme of time to grieve prior to the loss, due to the planned nature of Medical Aid in Dying, there is more “known” about the patient’s death.

The second overall theme identified was the place of death. One study^25^ reported participants stated they appreciated knowing that their family/friend had the opportunity to plan their death, specifically where they wanted to die. One study^3^ found similar results, as participants reported they were glad that Medical Aid in Dying allowed their family/friend to die at home, be in their own surroundings, and be around their family.

The third theme identified was a sense of control and autonomy for the person who engaged in Medical Aid in Dying. Two studies^27,29^ reported that participants felt that Medical Aid in Dying provided their family member/friend a sense of autonomy. Further, two studies^28,30^ found participants reported Medical Aid in Dying gave their family/friend the ability to schedule the day/time of their death, which provided the patient with a sense of control over the dying process. These studies concluded that allowing the patient to feel a sense of control and autonomy helped with the family member’s grieving process, as they knew the patient’s wishes were being honored.

The fourth theme identified was reducing suffering for the patient, which participants also noted assuaged the burden of watching the patient suffer. This theme was found in most of the qualitative studies (9 out of 10). For example, many studies^25,28,29^ reported that Medical Aid in Dying allowed them to avoid seeing the “body” of the patient decline and the family member did not have to see them suffer, which often accompanies the end stages of a terminal illness. Further, the participants in these studies stated it helped their grieving process, as they did not have to watch their family’s/friend’s quality of life decline. Many participants noted that, while grieving, they felt the fact their family/friend was not suffering and losing their abilities helped them through the process. Bereaved individuals also reported more time was a burden for everyone.^29^ More specifically, they reported the thought of the patient being alive longer (i.e. another 6 months) and watching them suffer was a burden on their relationship and the entire family unit. While not explicitly stated, this manner of death also indicates the caregiver was not engaged in physically caring for the individual while simultaneously trying to prepare and grieve the upcoming loss. Therefore, participants reported mitigating this burden through the patient’s engagement in Medical Aid in Dying reduced the family members’ negative bereavement outcomes.

The fifth theme identified was being able to support the wishes of the patient. One study^25^ reported participants stated the Medical Aid in Dying process allowed them to feel they were fulfilling the patient’s wishes. This included the patient’s vision for death and how the family member could help with that process (e.g. meditating with the patient; singing with the patient; giving the patient a massage; arranging who would be there at the end). Lastly, one study^27^ reported participants stated Medical Aid in Dying helped them cope with the loss, as they felt a sense of purpose in helping their family/friend with their end-of-life requests.

### Risk factors

There were six themes identified that were risk factors for negative bereavement outcomes following Medical Aid in Dying. These included moral problems, ambivalence toward Medical Aid in Dying, poor communication at the end of life, poor social integration and stigma, less time with the person with the terminal illness, and preparation for death. The first theme identified was moral problems with Medical Aid in Dying. One study^27^ stated the participants found it difficult to watch their family/friend take the medication and, while grieving, the participant began to fixate on the family/friend taking the medication. Second, in two studies, participants reported feelings of ambivalence about their family/friend engaging in Medical Aid in Dying both during the process and during bereavement.^26,28^ Participants described this as different than moral problems with Medical Aid in Dying, as they described it as not knowing if it was the right time or right for their family member to engage in. The third theme identified was poor communication at the end of life, which included communication between the family member and the patient as well as communication between the dyad and the medical provider. Studies^28,29^ reported participants stated the interaction with medical providers at the end of life regarding Medical Aid in Dying resulted in anger and frustration during bereavement. This included wanting to spend less time with medical providers, as some participants reported they felt their medical provider was heavily advocating for Medical Aid in Dying, while other participants reported their medical providers were trying to discourage or talk them out of engaging in Medical Aid in Dying.

Even though many of the studies discussed some of the themes (e.g. preparedness) as protective factors related to bereavement outcomes, Frolic et al.^29^ eloquently described many of the themes identified as a “double-edge experience,” as many of the themes identified during the Medical Aid in Dying process can confer both protection against and risk for negative bereavement outcomes. The final three themes would fall under this “double-edge experience.”

The first of the “double-edge experience,” and fourth overall theme identified was poor social integration and stigma following Medical Aid in Dying. These were mentioned in two studies,^29,31^ which reported that participants stated they perceived being stigmatized by others and felt they were not supported as much as individuals whose family/friend died a “natural death.” The fifth risk factor theme identified was less overall time with the person who engaged in Medical Aid in Dying. Participants reported that, following the loss, they felt they lost time with the person and “time is a gift”^30^ that was taken from them. The final theme, and another “double-edge” factor was the process of preparation for the death, as participants reported that preparation caused an emotional burden that started pre-death and continued into bereavement.^29^ More specifically, participants reported they were trying to take care of their family/friend while also grieving as they knew they were going to die soon resulting in an emotional burden and grief before the death.^13^

## Discussion

### Main findings/results of the study

This systematic review examined the extant literature on bereavement outcomes and specific risk and protective factors for bereavement outcomes of family members of individuals who had a family/friend participate in Medical Aid in Dying. Results indicated a paucity of studies investigating bereavement outcomes for this population. Moreover, there were vague findings regarding outcomes for family members of individuals who engage in Medical Aid in Dying compared to family members who lost someone due to other types of losses, including natural causes. Further, results revealed unique risk and protective factors for bereavement outcomes for this population that happen both before the death as well as after the death of the person who engages in Medical Aid in Dying. This highlights the importance of conducting longitudinal research to capture the risk and protective factors that start before the death in addition to those factors that may not onset until bereavement begins. Therefore, the theoretical model that we propose highlights risk and protective factors at each stage of the Medical Aid in Dying process (i.e. *pre-Medical Aid in Dying factors; post-Medical Aid in Dying Medical Aid in Dying factors*).

### What this study adds: Theoretical model

#### Pre-Medical Aid in Dying factors

**Figure 2.**
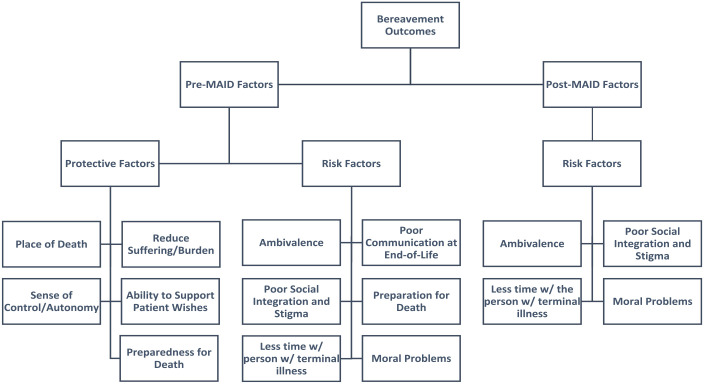
Theoretical model.

In synthesizing the study’s results, risk and protective factors were identified that occur following the terminal diagnosis but before the patient’s death (i.e. *Pre-Medical Aid in Dying factors*). Results indicated the risk factors were moral problems and ambivalence with Medical Aid in Dying, poor communication at the end of life, and preparation for the death. The protective factors included preparedness for death, place of death, sense of control and autonomy, reduced suffering and burden, and being able to support the patient’s wishes. See Figure 2 for the full model that describes *Pre-Medical Aid in Dying* risk and protective factors.

Interestingly, some of these risk factors (i.e. preparedness for death; place of death; reduce suffering/burden; poor communication at the end of life; poor social integration and stigma; preparation) have been identified in bereavement studies examining other types of losses (e.g. violent losses). For example, preparation for death has been noted to be a protective factor in numerous studies and systematic reviews^15,32^ regardless of cause of death. However, data synthesis revealed preparedness could be both a protective and a risk factor, whereas other studies have found it to be a robust protective factor for negative bereavement outcomes.^30,33^ One study identified in this systematic review^26^ found that preparedness was a protective factor, as participants stated preparedness for the death resulted in being able to say goodbye, allowing time to grieve before the loss, and feeling the loss was less of a shock. On the other hand, another study^29^ stated participants reported that preparation (i.e. knowing the date and time of death) caused an emotional burden for them that continued after the loss.^29^ Frolic et al.^29^ termed this the “double-edge experience,” as aspects of the Medical Aid in Dying process can be both protective and a risk factor for negative bereavement outcomes. Further research is needed to clarify the mechanisms underlying the bidirectional relations of these factors (e.g. preparedness) to understand their effect on bereavement outcomes. By gaining insight into the underlying mechanism, researchers can acquire a better understanding of whether preparedness, as described in non-Medical Aid in Dying studies as a protective factor,^34,35^ is the same construct/process as preparedness described in the articles identified in this systematic review.

It should be noted that many of the risk and protective factors (e.g. stigma; social integration)^36,37^ related to bereavement outcomes might differ in either intensity or underlying mechanism for a bereaved person whose family/friend engaged in Medical Aid in Dying. One study^36^ found stigma and poor social integration as both *Pre-Medical Aid in Dying* and *Post-Medical Aid in Dying* risk factors, which has not been found in family members of individuals in other types of losses (e.g. cancer; dementia). One reason these risk factors might lead to different outcomes for family members of individuals who engage in Medical Aid in Dying is due to the moral and ethical arguments surrounding Medical Aid in Dying. A family member, due to their own beliefs and/or beliefs held by their family and friends, might experience higher rates of self-stigma and public stigma as the patient starts the Medical Aid in Dying process. Further, this could contribute to negative downstream effects, as the family member might not receive adequate social support during the Medical Aid in Dying process.

#### Post-Medical Aid in Dying factors

*Post-Medical Aid in Dying* risk and protective factors were considered any risk or protective factor that occurs following the death of the patient who engaged in the Medical Aid in Dying process. Interestingly, there were fewer *Post-Medical Aid in Dying* risk factors in the literature and no protective factors from the studies identified in this systematic review, which highlights the need to conduct prospective studies examining risk and protective factors before the person has used the medication for Medical Aid in Dying. Even though there were no *Post-Medical Aid in Dying* protective factors identified in this systematic review there were numerous *Pre-Medical Aid in Dying* protective factors and studies are needed to examine *Post-Medical Aid in Dying* protective factors as many exist for physician aid in dying. The *post-Medical Aid in Dying* risk factors identified included poor social integration and stigma, moral problems and ambivalence with Medical Aid in Dying, and more time with the person with the terminal illness. As previously noted, stigma has been found to contribute to poor social integration, the latter of which is a robust predictor of worse bereavement outcomes (e.g. prolonged grief disorder).^38,39^ Research has aimed to understand stigma related to Medical Aid in Dying,^13^ but more research is needed to identify if perceived public stigma, and self-stigma, exist and their effects on bereavement outcomes for family members of individuals who experienced a Medical Aid in Dying related death.

Research discussed in this systematic review highlighted moral problems and ambivalence resulted in bereavement outcomes, as participants stated they “went back and forth” and “second guessed” their family member’s decision to utilize Medical Aid in Dying. This ambivalence or fluctuation when thinking about their family’s/friend’s death is not unique to this type of loss.^40,41^ For example, studies have found that some bereaved individuals engage in “second guessing” and “regret” related to how the person died, especially with violent loss (e.g. car accidents). The manner in which ambivalence manifests could differ in the context of Medical Aid in Dying, as the bereaved family member most likely played a critical role in the decision process, either supporting the person or helping them make the final decision to utilize Medical Aid in Dying medication. However, prospective, longitudinal research is needed to further understand family members’ feelings during the decision process, as retrospective measurements are subject to memory (e.g. Halo Effect) and social interaction biases,^34^ especially when coping is being reported retrospectively.

### Limitations

There were some limitations of the current study that must be discussed. First, most of the studies did not report the ethnic or racial breakdown of their participants, which limits our ability to understand possible risk and protective factor differences or similarities across cultural groups. Second, this systematic review only focused on articles that were published in English or for which translations were available. Third, we did not include conference abstracts, which could limit the studies identified. Fourth, we did not explore the impact of the delivery of the medication for Medical Aid in Dying (i.e. orally by themselves or by a healthcare provider) on the grief and bereavement process. This was predominately due to the reviewed articles not discussing the delivery of the medication’s impact on bereavement outcomes. Fifth, this study is limited by the multiple definitions used for “problematic” grief as prolonged grief disorder has only recently been added to DSM-5-TR and ICD-11. Therefore, as we are examining risk and protective factors identified in these articles our review is limited because of a lack of universal definition of “problematic” grief. Lastly, this systematic review summarized published results and did not attempt new analyses of the results nor the raw data underlying those results as most of the articles identified were mostly qualitative and the few (*n* = 3) quantitative articles used unique measures that could not be compared.

### Conclusions and future directions

This systematic review provides equivocal results about the effects of patients using Medical Aid in Dying on their family members following the loss of the patient and the risk and protective factors of bereavement outcomes. Nonetheless, the theoretical model built based on the results of this systematic review outlined some of the potential risk and protective factors. Future research should implement longitudinal methodology to investigate the translation of this theoretical model of risk and protective factors over time (i.e. pre-death into bereavement). In addition, examination of risk and protective factors that share a robust association with bereavement outcomes due to the type of loss should be conducted. For example, relationship satisfaction, pre-death depression, and worse emotional health prior to the patient’s diagnosis of the terminal illness leads to worse bereavement outcomes in other types of losses. However, these factors have not been investigated in studies on bereavement outcomes following Medical Aid in Dying, which limits our understanding of the pre-terminal diagnosis risk and protective factors for this population. These pre-terminal diagnosis factors could be important in predicting and implementing services for certain bereaved individuals who have lost someone to Medical Aid in Dying and may further extend the theoretical model outlined in the current study.

## Supplemental Material

sj-pdf-1-pmj-10.1177_02692163231172242 – Supplemental material for An examination and proposed theoretical model of risk and protective factors for bereavement outcomes for family members of individuals who engaged in medical aid in dying: A systematic review Click here for additional data file. Supplemental material, sj-pdf-1-pmj-10.1177_02692163231172242 for An examination and proposed theoretical model of risk and protective factors for bereavement outcomes for family members of individuals who engaged in medical aid in dying: A systematic review by Jonathan Singer, Courtney Daum, Amelia Evans, Sydnie Schneider, Margaret Vugrin and Elizabeth Loggers in Palliative Medicine
