# Supplementary material for: An examination and proposed theoretical model of risk and protective factors for bereavement outcomes for family members of individuals who engaged in medical aid in dying: A systematic review
Source: Palliat Med. 2023 May 2;37(7):947–58. doi: 10.1177/02692163231172242 (PMC10320704; doi:10.1177/02692163231172242)
Supplement: sj-pdf-1-pmj-10.1177_02692163231172242 – Supplemental material for An examination and proposed theoretical model of risk and protective factors for bereavement outcomes for family members of individuals who engaged in medical aid in dying: A systematic review [file sj-pdf-1-pmj-10.1177_02692163231172242.pdf]

Appendix A

2.3 Information Sources
A Texas Tech librarian experienced in conducting systematic reviews will conduct a search of each database independently:
Psycinfo
PsycARTICLES
CINHAL
PubMed
Sociological Abstracts
PsychiatryOnline
The Cochrane Library
EMBASE
SCOPUS
WebOfScience
2.4 Search Strategy

FIRST STEP
The databases will be searched for articles with the terms
"medical aid in dying," or "MAID," or "death with dignity," or "assisted suicide" or "assisted death" or medically assisted suicide" or "physician-assisted suicide" or "DWD" or "voluntary assisted dying" or "VAD" or "aid in dying" AND "end of life," or "depression," or "prolonged grief," or "prolonged grief disorder," or "complicated grief," or "grief," or "traumatic grief," or "bereavement," or "major depressive disorder," or "mental health," or "physician assisted suicide," or "terminal illness" or "post-traumatic stress disorder."

SECOND STEP
The bibliographies and references of included full-text articles and chapters will be reviewed to locate additional studies not found through database search and for additional keywords not included in the initial database search

THIRD STEP
Send out a call for in-press articles to (a) the first authors of identified studies, (b) identified grief experts/palliative care experts, and (c) to relevant list serves We will state in our email that authors have two weeks to respond before we will finish the manuscript with the current studies identified.

2.5 Study Records (i.e., Data Management; Selection Process; Data Collection Process)
Data Management: All data will be stored in a systematic review through Covidence. This will facilitate the screening process. A secure excel sheet will be used to categorize and characterize the final set of full-text articles.

Selection Process: Articles will first be assessed if they fit eligibility criteria. Second, reviewers will assess whether the study addresses mental health (e.g., grief; depression) in family members following a loved one's death due to MAID
